# Evolution of substrate specificity in bacterial AA10 lytic polysaccharide monooxygenases

**DOI:** 10.1186/1754-6834-7-109

**Published:** 2014-08-06

**Authors:** Adam J Book, Ragothaman M Yennamalli, Taichi E Takasuka, Cameron R Currie, George N Phillips, Brian G Fox

**Affiliations:** Department of Energy, Great Lakes Bioenergy Research Center, Madison, 1552 University Avenue, Madison, WI 53726 USA; Department of Bacteriology, University of Wisconsin-Madison, Microbial Sciences Building, 1550 Linden Dr., Madison, WI 53706 USA; Department of Biochemistry, University of Wisconsin-Madison, Biochemistry Addition, 433 Babcock Dr., Madison, WI 53706 USA; Biosciences at Rice, Rice University, George R. Brown Hall, Houston, TX 77005 USA

**Keywords:** Lytic polysaccharide monooxygenase, LPMO, Cellulase, Chitinase, Streptomyces, AA9, AA10, Enzyme evolution, Biofuels

## Abstract

**Background:**

Understanding the diversity of lignocellulose-degrading enzymes in nature will provide insights for the improvement of cellulolytic enzyme cocktails used in the biofuels industry. Two families of enzymes, fungal AA9 and bacterial AA10, have recently been characterized as crystalline cellulose or chitin-cleaving lytic polysaccharide monooxygenases (LPMOs). Here we analyze the sequences, structures, and evolution of LPMOs to understand the factors that may influence substrate specificity both within and between these enzyme families.

**Results:**

Comparative analysis of sequences, solved structures, and homology models from AA9 and AA10 LPMO families demonstrated that, although these two LPMO families are highly conserved, structurally they have minimal sequence similarity outside the active site residues. Phylogenetic analysis of the AA10 family identified clades with putative chitinolytic and cellulolytic activities. Estimation of the rate of synonymous versus non-synonymous substitutions (dN/dS) within two major AA10 subclades showed distinct selective pressures between putative cellulolytic genes (subclade A) and CBP21-like chitinolytic genes (subclade D). Estimation of site-specific selection demonstrated that changes in the active sites were strongly negatively selected in all subclades. Furthermore, all codons in the subclade D had dN/dS values of less than 0.7, whereas codons in the cellulolytic subclade had dN/dS values of greater than 1.5. Positively selected codons were enriched at sites localized on the surface of the protein adjacent to the active site.

**Conclusions:**

The structural similarity but absence of significant sequence similarity between AA9 and AA10 families suggests that these enzyme families share an ancient ancestral protein. Combined analysis of amino acid sites under Darwinian selection and structural homology modeling identified a subclade of AA10 with diversifying selection at different surfaces, potentially used for cellulose-binding and protein-protein interactions. Together, these data indicate that AA10 LPMOs are under selection to change their function, which may optimize cellulolytic activity. This work provides a phylogenetic basis for identifying and classifying additional cellulolytic or chitinolytic LPMOs.

**Electronic supplementary material:**

The online version of this article (doi:10.1186/1754-6834-7-109) contains supplementary material, which is available to authorized users.

## Background

The two most abundant polysaccharides in nature are cellulose and chitin [1]. Plants, insects, crustaceans, molluscs, and fungi all utilize these two highly stable polymers as primary components of their cell walls. Deconstruction of polysaccharides is essential for ecosystem-level carbon and nitrogen cycling. Moreover, polysaccharides are potential energy sources that could help supplement the current massive demand for fossil fuels [2]. Intensive efforts worldwide focus on conversion of these energy-rich biomolecules into free sugars that can be fermented into biofuels or other value-added bioproducts. However, hydrolysis of these polymers is difficult due to their crystalline structure, the stability of the β-glucosidic bond, and their close association with hemicellulose, lignin, and other modifying molecules [1, 3]. Cellulolytic and chitinolytic enzymes capable of this have been identified in a myriad of organisms, but most often in bacteria and fungi [4]. While the biochemical activities and mechanisms of hydrolytic enzymes have been known for decades, oxygenolytic pathways for deconstruction of chitin and cellulose have only recently been identified [5–8].

CBH1, one of the first representatives of what are now recognized to be lytic polysaccharides monooxygenases (LPMOs), was secreted by *Streptomyces olivaceoviridis* and interacted with α-chitin, but since it lacked classical hydrolytic activity, it was thus considered to be a non-hydrolytic carbohydrate binding module (CBM) [9]. An ortholog of CBH1, chitin-binding protein 21 (CBP21) was identified in *Serratia marcescens* [10] and initially classified as carbohydrate binding module 33 (CBM33, now systematically called Auxiliary Activity 10, AA10).^a^ The function of CBP21 was first demonstrated by Vaaje-Kolstad *et al*. [11], who showed cleavage of crystalline chitin in an O_2_-dependent reaction. Soon after this report, others showed that the eukaryotic counterpart, fungal glycoside hydrolase 61 (GH61, now systematically called Auxiliary Activity 9, AA9) was a Cu^2+^-dependent enzyme [11–14]. An oxidative function has also been demonstrated for CelS2, an AA10 from *Streptomyces coelicolor* [6], which reacts synergistically with hydrolytic cellobiohydrolases and endoglucanases [15], and more recently for BlAA10A from *Bacillus licheniformis* and E8 from *Thermobifida fusca* [16] which react with chitin and cellulose, respectively, giving four AA10 enzymes whose function has been determined.

AA9 and AA10 incorporate a single ^18^O from ^18^O_2_ into polysaccharide cleavage products, and so are now classified as LPMOs [11]. To date, structures of six AA9 and five AA10 enzymes have been solved, including one nuclear magnetic resonance (NMR) structure [11, 17–19]. Overall, the two LPMO families share a conserved β-sandwich fold [11], and many residues on the substrate-binding surface are conserved. Moreover, Cu^2+^ has been identified in the active sites [8, 17, 18]. Although recent computational studies support the involvement of a copper-oxyl radical intermediate [20], the catalytic mechanism of this reaction is still largely unexplored.

Oxidative polysaccharide cleavage results in the formation of an aldonic acid from C1 oxidation [21] or a ketoaldose from C4 oxidation [21, 22]. The monooxygenase reaction stoichiometry requires the addition of 2e^-^ from an oxidoreductase or other external electron donor. The presence of oxidoreductases has been reported in various cellulolytic fungi [23], though an actual, physiological electron partner for LPMOs has not been unambiguously determined.

In this study, we compared amino acid sequences and protein structures in order to explore the evolutionary relatedness of AA9 and AA10. Conserved sequence and structural features were correlated with potential substrate interactions and surfaces potentially used by electron donors. Phylogenetic analysis suggests that cellulose- and chitin-specific enzymes are distributed into different subclades within bacterial AA10, as has been recently reported for the fungal AA9 [18, 21]. Potential evolutionarily pressures within the AA10 family were examined in order to understand how Darwinian selection might have influenced substrate specificity.

## Results

### Structural comparison of LPMO families AA9 and AA10

**Figure 1:**
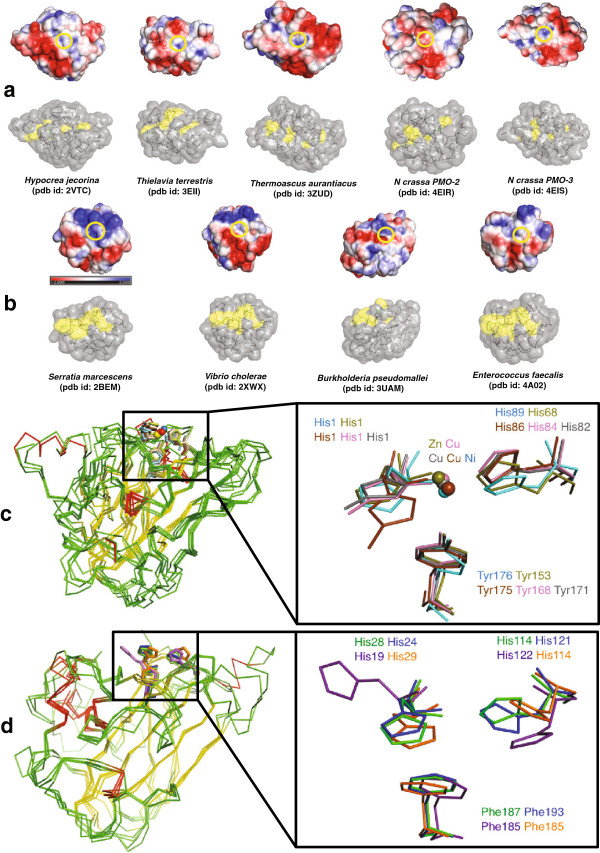
**Electrostatic surface comparison between AA9 and AA10 lytic polysaccharide monooxygenases.** The images show the protein surface containing the active site as presented to the substrate. The yellow circles indicate the location of the catalytic residues and bound Cu^2+^. **(a)** The AA9 family has a strip of positively charged surface sandwiched in an overall negatively charged surface (shown in red). **(b)** In the AA10 family, a patch of positively charged surface (shown in blue) is adjacent to the active site. **(c)** Superposition and inset showing the active site residues of AA9 from *Hypocrea jecorina* (pdb id: 2VTC), *Thielavia terrestris* (pdb id: 3EII), *Thermoascus aurantiacus* (pdb id: 3ZUD), and *Neurospora crassa* (pdb id: 4EIS and 4EIR) in ribbon representation and colored with respect to secondary structure (helix-red; strand-yellow; loop-green). The residues involved in the active site are shown as sticks and colored blue for *H. jecorina*, magenta for *T. terrestris*, orange for *T. aurantiacus*, and light brown and gray for *N. crassa*. The divalent metal atoms (Ni, Zn, Cu) are shown as spheres. The active site residues labeled in the inset are colored the same as the intact structures. **(d)** Superposition and inset showing the active site residues of AA10 from *Serratia marcescens* (pdb id: 2BEM), *Vibrio cholerae* (pdb id: 2XWX), *Burkholderia pseudomallei* (pdb id: 3UAM), and *Enterococcus faecalis* (pdb id: 4A02) shown in ribbon representation and colored with respect to secondary structure. The residues (inset) involved in the active site are shown as sticks and colored green for *S. marcescens*, blue for *V. cholerae*, magenta for *B. pseudomallei*, and orange for *E. faecalis.*

Figure 1a shows five crystal structures from the AA9 family. These are from *Hypocrea jecorina* (*Trichoderma reesei*, Protein Data Bank (pdb) id: 2VTC) [24], *Thielavia terrestris* (pdb id: 3EII) [7], *Thermoascus aurantiacus* (pdb id: 2YET) [13], *Neurospora crassa* PMO-2 (pdb id: 4EIR) [18], and *N. crassa* PMO-3 (pdb id: 4EIS) [18]. Structures of four AA10 enzymes are also shown in Figure 1b. These are from *S. marcescens* (pdb id: 2BEM) [25], *Vibrio cholerae O1 biovar EI Tor* (pdb id: 2XWX) [26], *Burkholderia pseudomallei* (pdb id: 3UAM), and *Enterococcus faecalis* (pdb id: 4A02) [27]. Both AA9 and AA10 have a conserved β-sandwich fold with three to four β-sheet strands (Figure 1c and 1d). The average root mean square (RMS) deviation of the aligned structures is approximately 3 Å (Table 1) In addition to the fold-level similarity between AA9 and AA10, two key histidine (His) residues that coordinate a Cu^2+^ ion at their active sites are also highly conserved in both families (Table 1 and Figure 1c). The structural superposition of the metal ligands suggests that this configuration is essential for activity (inset in Figure 1c and 1d). A notable difference between AA9 and AA10 is the third, non-coordinating active site residue; being primarily tyrosine in the former and primarily phenylalanine in the latter, with a relatively few exceptions presently also identified.

**Table 1:** **Structural homology of lytic polysaccharide monooxygenases**

PDB ID	% RMSD ^1^	%id	Source structure	CAZy family	Source organism	Active site residues
2BEM	0	100	X-ray	AA10	*Serratia marcescens*	H28-H114-F187
2XWX	0.8	51	X-ray	AA10	*Vibrio cholerae*	H24-H121-F193
4A02	1	52	X-ray	AA10	*Enterococcus faecalis*	H29-H114-F185
2LHS	1.4	100	NMR	AA10	*Serratia marcescens*	H28-H114-F187
3UAM	1.4	39	X-ray	AA10	*Burkholderia pseudomallei*	H19-H122-F205
2VTC	3.2	9	X-ray	AA9	*Hypocrea jecorina*	H1-H89-Y176
4EIR	2.8	9	X-ray	AA9	*Neurospora crassa*	H1-H84-Y168
3ZUD	3.3	12	X-ray	AA9	*Thermoascus aurantiacus*	H1-H86-Y175
3EII	3.2	11	X-ray	AA9	*Thielavia terrestris*	H1-H68-Y153
4EIS	2.8	7	X-ray	AA9	*Neurospora crassa*	H1-H82-Y171

^1^Root mean square deviation (RMSD) (%) for each structure from Protein Data Bank (pdb) compared to 2BEM determined by X-ray crystallography; %id indicates the percentage identity of each sequence to that of 2BEM. Three active site residues, His, His, and Phe/Tyr, are shown with residue numbers.

Six AA10 structures from *E. faecalis* released in the pdb show copper in the active site, and a recently published structure of AA9 from *Phanerochaete chrysosporium* (pdb id: 4B5Q) also shows copper bound in the active site [28]. Copper binds with nanomolar affinity to AA10 [8, 17]; its presence is consistent with O_2_ activation required for the LPMO reaction.

### Surface electrostatic potential on the binding surfaces of AA9 and AA10

To explore factors that may contribute to substrate specificity in the AA9 and AA10 families, we characterized the electrostatic potential present at the substrate-binding surface. In both families, the metal-binding histidine residues are part of a planar surface that constitutes the polysaccharide-binding surface [18]. Figure 1a and 1b show the surface electrostatic potential of representatives from both AA9 and AA10 families. For the AA9 proteins, which are biochemically characterized as cellulose monooxygenases, negatively charged residues (shown in red) prominently surround the active site (Figure 1a, yellow circle). In contrast, the AA10 chitin monooxygenases contain both positively charged (shown in blue) and negatively charged residues (shown in red) surrounding the active site (Figure 1b, yellow circle). Aachmann *et al.* [17] used NMR to identify residues from the chitinolytic AA10 enzyme from *S. marcescens* (pdb id: 2BEM) that are involved in chitin binding. These residues are Q53, Y54, E55, Q67, S58, L110, T111, A112, H114, and T116 [17]. The positions of the corresponding residues from the other AA10 enzymes that align with 2BEM are shown as yellow on a grey surface in the lower parts of Figure 1a and 1b. In the other members of the AA10 family, most of these structurally conserved residues are also surface-exposed (Figure 1b, bottom). However, in the AA9 family, only a few are exposed at the polysaccharide-binding surface (Figure 1a, bottom), indicating that different residues from the folded structures will be involved in substrate binding in the AA9 and AA10 families.

### Diversity of domain structures in AA9 and AA10 proteins

**Figure 2:**
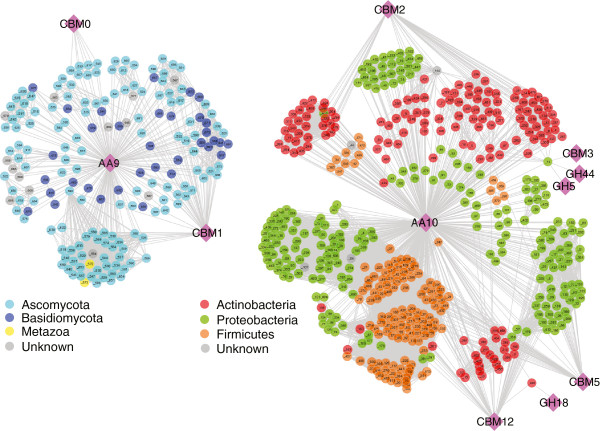
**Domain and sequence similarity networks for the LPMO superfamily.** Circles represent proteins from either the AA9 or AA10 families, diamonds represent CAZy annotations. Edges represent either BLAST similarity with a bit score greater than 200 (evalue > e^-50^) or annotation to the indicated CAZy functional group. Colors represent taxonomic distribution of phyla of the source organisms. No sequence similarity above the indicated threshold was identified between the AA9 and AA10 superfamilies.

Another possible determinant of substrate specificity within the AA9 and AA10 families is the domain architecture. LPMO enzymes have a diverse composition of domains: they can be single catalytic domains, associated with various CBMs, or even associated with other catalytic domains (such as glycoside hydrolase (GH) domains). Figure 2 shows a Cytoscape (The Cytoscape Consortium, San Diego, CA) protein sequence homology network accounting for the variations in domains in the AA9 and AA10 families, where nodes represent enzymes or functional classes, and edges represent sequence similarity (bit score >200, evalue <1e^-50^). In order to prepare this network, sequences were collected from CAZy, compared via pairwise BLAST analysis, and then annotated with secondary CAZy domains. Nodes are colored according to their phylum-level taxonomic identification. The network contains 184 AA9 sequences and 495 AA10 sequences. All AA9 proteins were from eukaryotes, with a vast majority (99%) from the fungal phyla Ascomycota (135 sequences) and Basidiomycota (34 sequences). Of the protein sequence in the AA9 family, 31% include a secondary carbohydrate binding module 1 (CBM1), which has been reported to bind cellulose [29]. Seven AA9 sequences are associated with CBM0, an unclassified CBM family [30, 31].

The AA10 family is exclusively from prokaryotes, with 226 sequences from Proteobacteria, 145 from Actinobacteria, and 132 from Firmicutes (Figure 2). There were no edges linking members of the AA9 and AA10 families at the similarity threshold of evalue <1e^-50^. Furthermore, when the similarity threshold was relaxed to 1e^-5^ there were still no connections between the AA9 and AA10 families. While Figure 2 shows that the AA9 network contains interspersed sequences from Ascomycota and Basidiomycota, the AA10 family shows clear taxonomic groupings assembled from different bacterial phyla. These results also show that while the active site residues of the AA9 and AA10 families are mostly conserved (Table 1), these two families do not share any other significant sequence similarities or consistent linkages to other domains.

Figure 2 also shows that the AA10 family is combined with a variety of secondary CBM domains, with 31% of the total sequences including cellulose-binding domains CBM2 and CBM3 [32, 33] or chitin-binding domains CBM5 and CBM12 [34]. Further phylogenetic binning of AA10 showed expansion within the genera of *Streptomyces*, *Bacillus*, and *Vibrio* (Additional file 1: Figure S1). Interestingly, 94% of the AA10 sequences that included a cellulose-binding CBM were from the phylum Actinobacteria, whereas 95% of sequences including a chitin-binding CBM were from the phyla Firmicutes and Proteobacteria. Finally, two genes were identified that also encoded a glycoside hydrolase domain, suggesting a rare but possibly synergistic pairing of glycoside hydrolase and LPMO catalytic activities in a single enzyme.

### Phylogenic analysis of LPMO families

**Figure 3:**
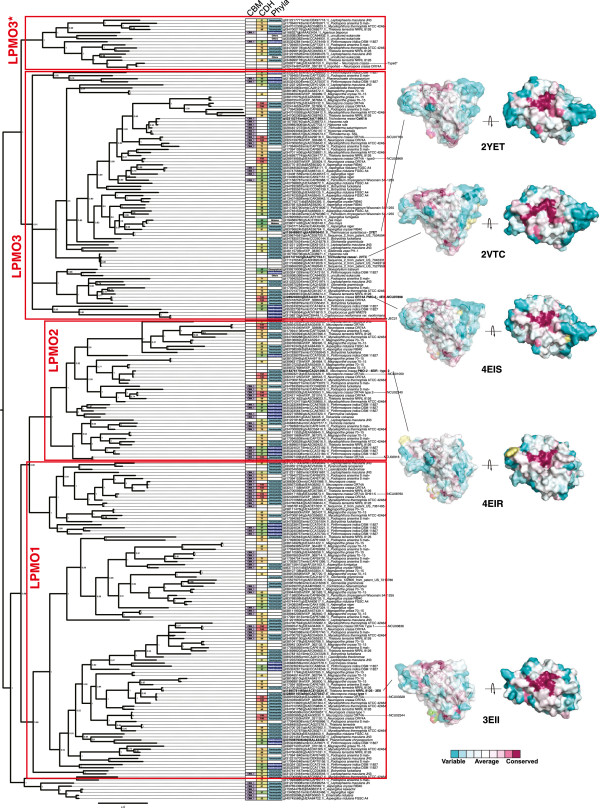
**Phylogenetic analysis of the AA9 LPMO superfamily.** MrBayes phylogenetic tree for 254 AA9 protein sequences. The tree was generated using the catalytic domain of the AA9 protein only. Additional carbohydrate-binding domains that are present in the full protein sequence are indicated in the CBM column, but were not included in the calculation of the tree structure. Source organisms were searched for the presence of a homolog to *Neurospora crassa* cellobiose dehydrogenase (CDH1). Protein identity scores are indicated in the CDH column, and colors range from 30% identity (green) to 100% identity (red). Solved structures have been mapped onto the tree and colors represent conservation of residues across the whole AA9 family.

**Figure 4:**
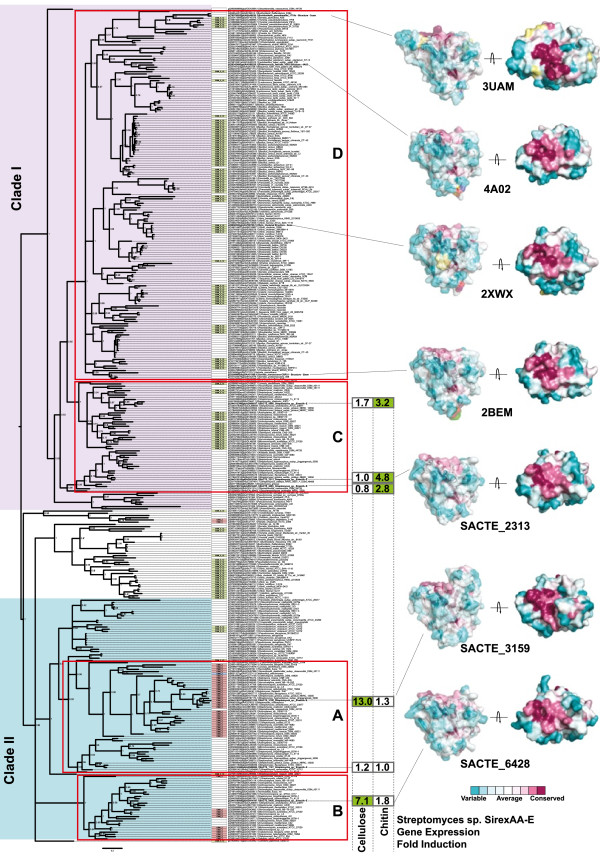
**Phylogenetic analysis of the AA10 LPMO superfamily.** MrBayes phylogenetic tree for the 374 AA10 protein sequences. The tree was generated using the catalytic domain of the AA10 protein only. Additional carbohydrate-binding domains that are present in the full protein sequence are indicated in the CBM column, but were not included in the calculation of the tree structure. Solved structures have been mapped onto the tree and colors represent conservation of residues across the whole AA9 family, and in the three modeled structures for *Streptomyces* sp. SirexAA-E and AA10 enzymes. Gene expression data for the six AA10 isoforms from SirexAA-E showing fold change in transcripts from glucose grown cells to either cellulose or chitin grown cells.

To gain further insight into the evolutionary relationship and possible functional roles of the distinct LPMO families, we created phylogenetic trees representing the AA9 and AA10 families (Figures 3 and 4, respectively). Briefly, sequences were collected, curated to remove redundant sequences with 100% identity, aligned, trimmed to the conserved catalytic domain, and then the tree was constructed by MrBayes phylogenetic analysis [35]. The resulting consensus tree was midpoint rooted and annotated with associated carbohydrate-binding modules in addition to the AA9 or AA10 catalytic domains. The five crystal structures determined for AA9, 2YET, 2VTC, 4EIS, 4EIR, and 3EII, were mapped onto the phylogenetic tree. In Figure 3, the surfaces of these structures have been colored to identify highly conserved residues shared across the AA9 family. The tree was also annotated to indicate whenever a putative cellobiose dehydrogenase (AA3 family enzymes) was present in the host genome using a cutoff criterion of 35% identity to *N. crassa* CDH1. The ability of CDH to act as the proximal electron donor for LPMO in cellulose oxidative cleavage has been demonstrated in this organism [18, 36–38].

The AA9 LPMOs have been classified into four functional types based on their reaction products [21]. These are shown in Figure 3 as red boxes. LPMO1 enzymes hydroxylate the C1 position of pyranose rings and produce an aldonolactone [18, 21], while LPMO2 enzymes hydroxylate the C4 position of pyranose rings and produce a 4-ketoaldose [21, 22]. LPMO3 enzymes are less specific [13, 19, 21, 39], and produce both aldonolactone and non-reducing end oxidized products, while LPMO3* produce only aldonic acids [21].Mapping of the four LPMO subgroups onto the global AA9 phylogeny showed that the LPMO2, LPMO3, and LPMO3* subgroups are monophyletic, with each having a single phylogenetic clade that corresponds to distinct functional classes (red boxes). In contrast, LPMO1 enzymes span a major evolutionary division as two branches cross into this functional class, indicating more sequence diversity in the LPMO1 family. Examples where all four LPMO functional types were fused to additional CBM domains are identified in Figure 3. Moreover, Figure 3 also shows that the majority of AA9 proteins come from organisms that also contain a cellobiose dehydrogenase homolog.

The AA10 phylogenetic tree was generated in a similar manner using the catalytic domains of all non-redundant sequences present in the CAZy database. The AA10 tree shown in Figure 4 represents 374 non-redundant sequences that are entirely bacterial in origin. The tree was annotated with secondary CBM domains (central column), and divided into two major clades (clade I and clade II) that could be subdivided into four additional subclades (A through D). The biochemically characterized cellulose-oxidizing LPMOs from *S. coelicolor* (A3) and *T. fusca* are present in subclade A,^b^ while all other LPMOs with experimental confirmation of their reaction with chitin are present in subclades C and D [6].The tree was also annotated with microarray-based gene expression data for the six variants of AA10 present in *Streptomyces* sp. SirexAA-E (SirexAA-E) [40]. Clade I contains a delineated mixture of phyla, with subclade C containing sequences only from Actinobacteria and with subclade D containing sequences from Firmicutes and Proteobacteria. Clade II is primarily composed of Actinobacteria and separates into subclades A and B. Subclades A and B contain only cellulose-binding CBMs (CBM2 and CBM3) associated with the catalytic AA10 domain, whereas subclades C and D contain only chitin-binding CBMs (CBM5 and 12). Furthermore, expression data from SirexAA-E shows that genes from subclades A and B were selectively upregulated only when cells were grown in medium containing cellulose as the sole carbon source, while genes from subclade C were upregulated only during growth on chitin [40].

The cellulose-oxidizing LPMOs from AA10 are primarily present in Actinobacteria, an aerobic filamentous bacterial phyla found in soil, but also associated with insects and other animals [40]. In Figure 4, the structures of four AA10 enzymes are mapped to the phylogenetic tree: 3UAM, 4A02, 2BEM, and 2XWX. Additionally, predicted protein structures for expressed AA10 from SirexAA-E are mapped onto the tree. There is high amino-acid sequence identity among the AA10 proteins whose structures have been determined, with the highest sequence conservation observed at the active site (magenta color). Interestingly, homology models consistently predict an additional surface exposed loop region on the same side of the protein as the active site in clade II proteins (chitin oxidation), but not in clade I (cellulose oxidation). The position of this loop can be recognized in pdb id: 4GBO, the E7 enzyme from *T. fusca* [16] *.* Recently, Vu *et al*. have identified a role for these extra loops in substrate recognition and control of specificity of reaction in the AA9 family [21].

### Homology modeling of AA10 proteins and conserved sequence motif in LPMOs

**Figure 5:**
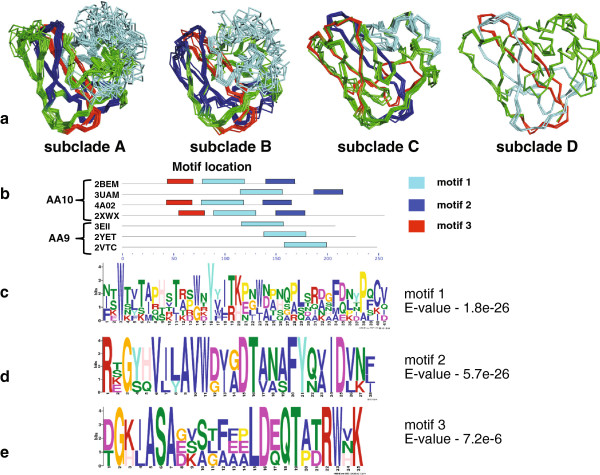
**Three Multiple Em for Motif Elicitation (MEME) motifs mapped to the predicted structures of AA10.** MEME was used to identify motifs from four separate subclades A to D from the phylogenetic tree of AA10. The loops are shown for illustration purposes only. The number of sequences in each clade is as follows: subclade A (68 sequences), clade B (29 sequences), clade C (77 sequences), and clade D (122 sequences). These motifs were mapped to the structures that were predicted using iterative threading assembly refinement algorithm (I-TASSER), as shown in **(a)**. Motif1 is shown in cyan color, motif2 is shown in blue and motif3 is shown in red. **(b)** MEME motifs mapped to the sequences from the available crystallographic structures of AA10 and AA9, showing the distribution of three motifs in the sequence. Sequence logo of **(c)** motif1 (cyan), **(d)** motif2 (blue), and **(e)** motif3 (red) in the available structures.

Several LPMOs from within the AA10 family have been experimentally verified to be either chitin or cellulose monooxygenases (such as CBP21 and BlAA10A) which react with chitin, and CelS2 and E8, which react with cellulose [6, 25].^b^ To further explore structural determinants that control substrate specificity, we compared homology models for 43 proteins that spanned the AA10 family (Figure 5) across the clade I and clade II sequences shown in Figure 4. Homology modeling using I_TASSER [41], followed by superposition of the modeled structures showed that the most significant structural differences were located in the substrate binding region (Figure 5, Additional file 2: Table S1). Specifically, the positions of loops (shown for illustration purposes only) on the substrate-binding side of the protein had more variations than other parts of the modeled structures. Correspondingly, the insertion observed in the sequence alignments mapped to loops on the substrate-binding side of the AA10 family. Given the structural variability of clades I and II, and differences in measured catalytic functions, it is likely that these structural differences help to modulate substrate selectivity between chitin and cellulose in AA10, as now predicted for AA9.

To improve our mapping of potential functional determinants onto the modeled structures, Multiple EM for Motif Elicitation (MEME) [42] was used. This approach identified three sequence motifs among the 43 AA10 proteins (Figure 5). These motifs were mapped back onto the structures and homology models. Simultaneously, MEME was used to determine whether there were significant motifs observed in the published structures of AA9 (Figure 5).In the homology-modeled proteins (shown in Figure 5 corresponding to the four AA10 clades shown in Figure 4), the three MEME motifs ranged from about 25 to 41 residues in length. Motif1 was present in both AA9 and AA10, and contained the variable insertion regions that possibly yield substrate selectivity in the AA10 family. Motif2 and motif3 were observed only in AA10 (Figure 5b). It is interesting to note that the difference in the number of motifs identified in AA9 as compared to AA10 provides an additional line of evidence supporting the possibility of evolutionary selection in these two families. Although experimental evidence as to what these motifs (Figure 5c-e) contribute is currently lacking, it is clear from the superposition of the homology-modeled structures (Figure 5, cyan sequences) that motif1 is well-positioned to play a role in substrate binding and discrimination between binding to chitin or cellulose. Interestingly, motif2 and motif3 span the breadth of the protein and connect the substrate-binding surface to the opposite side of the protein where potential electron donor proteins might interact.

### Evolution of chitinolytic and cellulolytic subclades within the AA10 family

**Figure 6:**
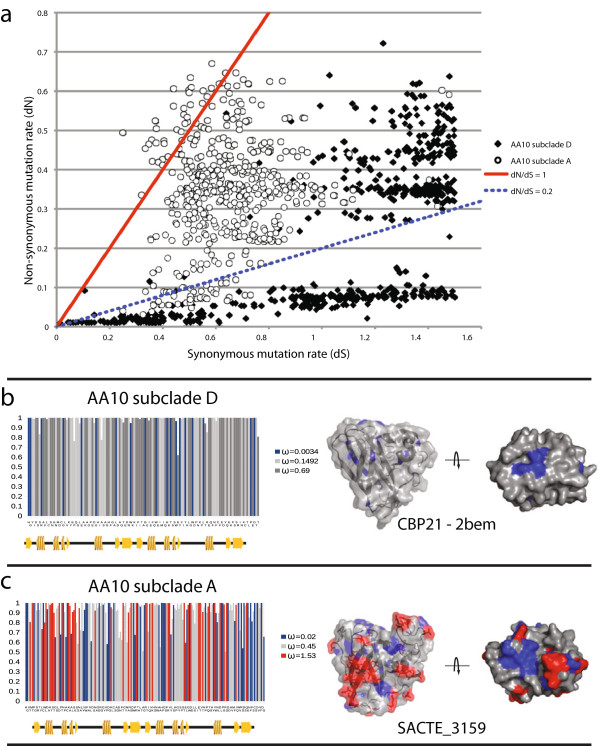
**Evolution of chitinolytic and cellulolytic AA10 genes. (a)** Plot of pairwise estimated dN and dS for genes from cellulolytic and chitinolytic clades, clade A (circles) and clade D (diamonds), respectively. Values were calculated by pairwise comparison of all genes within each clade and filtered to remove insignificant values (see text). Also shown are trend lines for dN/dS ratios of 1 and 0.2, representing approximate thresholds for positive and negative selection. Site-specific estimation of dN/dS ratios for clade A **(b)** and clade D **(c)**. Positively selected residues are colored in red, neutral in grey, and negative in blue (x-axis corresponds to protein sequence and y-axis to the posterior probability of the estimation). dN/dS ratios were also mapped onto the CBP21 structure 2BEM, and the modeled structure for SACTE_3159. Colors correspond to selection rates described above.

To evaluate the selective pressure on these functionally defined clades, the rates of non-synonymous and synonymous codon substitutions (dN and dS, respectively) of the catalytic domain were estimated (Figure 6a). Pairwise comparisons were performed against all genes within either subclade A or subclade D. Estimations with dN values greater than 0.01 and dS values less than 1.5 were reported to allow sufficient mutational signal and to avoid the effect of back mutations that would artificially increase dS and reduce dN with increased sequence divergence [43]. Pairwise comparisons indicate that a group of the chitinolytic genes from subclade D are primarily under negative selection (Figure 6, dN/dS <0.2), while a second group is under more neutral selection (1 > dN/dS >0.2). However, genes from subclade A have a significantly different distribution than from subclade D. Very few genes from cellulolytic subclade A show negative selection, while a significant proportion show increased positive selection (dN/dS >1). To confirm these results, site-specific dN/dS values were estimated for subclades A and D (Figure 6b). The results show that a significant number of residues in subclade A were indeed positively selected, while residues in subclade D were all negatively or neutrally selected. When plotted on the protein structures, the negatively selected sites in both subclades A and D are primarily located around the active-site residues. In contrast, most of the positively selected residues in subclade A are surface exposed, including regions on the putative substrate-binding surface and along the interior of the protein traversing from the substrate-binding surface to the opposite surface of the protein. Interestingly, this latter region may provide a surface for interaction with accessory redox proteins such as cellobiose dehydrogenase (AA3 enzymes).

## Discussion

In this study, we analyzed the AA9 and AA10 families using available protein structures and sequence information to evaluate differences between and within the families, to explore features that influence substrate specificity, and to characterize selective pressures that may have led to functional diversification.

LPMOs share a common structural fold and a spatial conservation of active site residues, as seen by their low root mean square deviation (RMSD) values (ranging up to 3.3 Å, Table 1). While the core structural folds and the active site geometry of these two LPMO families are similar, there is low homology at the amino-acid sequence level, and the surface electrostatic potentials at the substrate-binding surface show considerable differences in charge distributions. Indeed, comparison of all AA9 and AA10 proteins available in the CAZy database failed to identify any sequences from across these two families that have significant homology (evalue <1e^-5^). Our results indicate that although AA9 and AA10 families share structural similarities, they have so significantly diverged from a common ancestor that the only residue-level homology that remains is in the active site residues.

Due to the low sequence similarity between AA9 and AA10 families we analyzed their phylogenetic relationships separately. The AA9 phylogenetic tree is separated into three major evolutionarily related groups which partially correspond to the four types of enzyme activity observed for LPMOs [21]. LPMO2, LPMO3, and LPMO3* enzyme activities correspond to monophyletic clades, which suggests vertical inheritance and conserved enzyme functions within each clade. In contrast, LPMO1 enzymes are present in a polyphyletic clade, indicating a more diverse sequence space and potentially varied enzyme function. Sequences from Ascomycetes and Basidiomycetes are scattered throughout the three major evolutionarily related groups in AA9, suggesting an ancestral sequence that was shared before these two phyla separated.

The AA10 phylogenetic tree was separated into two major phylogenetic groups. When annotated with known activities, the two clades appear to separate enzymes with different substrate specificities. Clade I contains all biochemically defined chitin monooxygenases, while clade II contains subclades that are either cellulose or chitin monooxygenases. Gene expression data from SirexAA-E grown on either chitin or cellulose as the sole carbon source further corroborates this assessment [40]. We also observed that CBM domain composition varies between clade I and II. Clade I is dominated by CBM5 and 12 domains, which are primarily chitin binding, but possibly can have a lignin-binding function as well [44]. Clade II is enriched in CBM2 domains, which are primarily associated with cellulose binding. Most recently, Forsberg *et al*. showed the binding specificity of CelS2 either with or without the associated CBM2 domain [16]. Interestingly, although this CBM2 domain was tightly bound to either α or β-chitin, the corresponding AA10 domain (CelS2) only reacted with cellulose. Further biochemical verification will be necessary to extend these observations more broadly into phylogenetic space.

To identify sequence and structural features that may contribute to clade II activity against cellulose, we generated homology-modeled structures for 43 sequences that span the phylogeny in AA10. Using MEME, these homology-modeled structures were identified to have three highly significant motifs, where motif1 shows the largest structural variability. Specifically, this variable motif is contained in a loop of un-modeled sequence at the substrate-binding surface and is only found in subclade A. Subclade A of AA10 contains biochemically characterized cellulose monooxygenases, and also contains the most highly upregulated AA10 enzyme when SirexAA-E is grown on cellulose [40]. We hypothesize that this additional sequence at the binding surface is a defining feature of cellulose-active AA10 enzymes, paralleling the identification of a loop-modulating reaction specificity in the AA9 enzymes [21]. Motif2 and motif3, which span the breadth of the protein, connect the substrate-binding surface to the opposite side of the protein. This suggests a possibility for modulation of electron donor interactions.

Finally, we explored the selective pressures within two clades of the AA10 family to understand how diversification may be distributed in this enzyme family. The results show that chitinolytic enzymes in subclade D (chitinolytic enzymes) have mostly negative selection at both the whole gene and site-specific levels. In contrast, subclade A (both chitinolytic and cellulolytic enzymes) contains more genes with diversifying selection at both the whole gene and site-specific levels. This result indicates that subclade A may have undergone a change in substrate specificity and that genes within this clade are potentially being selected for increased activity. Together, these data suggest that the ancestral form of AA10 may have been a chitin monooxygenase, and that clade II has apparently further specialized for cellulose oxidation. Selection may be towards more favorable substrate binding, better interactions with accessory redox proteins, such as cellobiose dehydrogenase enzymes, or perhaps both.

## Conclusions

In summary, this study provides a better understanding of the evolution of functional diversity within the recently discovered AA9 and AA10 LPMO families. Together, these data suggest that AA9 and AA10 families share a distant common ancestor. Furthermore, clades within the AA10 family are specialized for different substrates and subclade A has undergone diversifying selection at surface-exposed regions of the protein.

## Materials and methods

### Sequence similarity network

AA9 and AA10 protein-coding sequences were identified on the Carbohydrate-Active Enzyme (CAZy) database [45], and harvested from the National Center for Biotechnology Information (NCBI) protein database. All AA9 and AA10 sequences were compared against each other using BLAST [46] to identify similar proteins. All sequences were also re-annotated with CAZy families to identify the domain structure of each protein. This data was then used to build a similarity network using Cytoscape 2.8.0 [47], and visualized as an organic layout. Nodes in the network represent unique protein sequences and CAZy families. Edges represent a BLAST bit score of ≥200 (evalue ≥1 × e^-50^), or an annotation to a CAZy category. Nodes were annotated with taxonomic information at the phylum level.

### Phylogenetic tree construction

AA9 and AA10 phylogenetic trees were constructed by first identifying proteins from the CAZy database, and the harvesting sequence from NCBI. Sequences from either AA9 or AA10 families were aligned using Multiple Sequence Comparison by Log-Expectation (MUSCLE) on the Cyberinfrastructure for Phylogenetic Research (CIPRES, https://www.phylo.org/portal2/login!input.action) Science Gateway [48]. Aligned sequences were then trimmed to retain only the AA9 or AA10 domain; sequences lacking the conserved active site His residues were removed from the alignment. Phylogenetic trees were generated using MrBayes code with a calculated standard deviation of ≤0.05. Non-default parameters were set to mcmc, ngen = 10,000,000, temp = 0.200, burninfrac = 0.25, stoprule = No, sump burnin = 4000, and sumt burnin = 4000. Resulting trees were annotated with pfam, phyla, solved structures, and cellobiose dehydrogenase homolog information.

### Evolutionary rate estimation

Coding sequences for subclades A-D from the AA10 family were collected and codon alignments were generated with MUSCLE. Sequences were trimmed to retain only the AA10 domain. Codon alignments were masked with Zorro (http://phylogenomics.wordpress.com/software/zorro/) to generate quality scores for codon positions [49], and then a phylogenetic tree was generated with RAxML (http://sco.h-its.org/exelixis/web/software/raxml/index.html) using the masking scores [50]. Pairwise codon substitution models (dN/dS values) were estimated using the CODEML program in the PAML package [51, 52]. Variables were set at CodonFreq = 0 and model = 0. Only pairwise dN values with values ≥0.01 and dS values ≤1.5 were reported so as to allow for sufficient mutational signal and to avoid the effects of back mutations. Site-specific codon substitution models were generated using the CODEML program in PAML, with model = 0, NSsites = 3, ncatG = 3 fix_kappa = 0, fix_omega = 0, cleandata = 1, and fix_blength = 2.

### Protein three-dimensional structure comparison

The Dali protein structure alignment database (http://ekhidna.biocenter.helsinki.fi/dali_server/) was used to calculate %RMSD and %ID of LPMO enzymes whose structures are known using 2BEM as a query [53]. Structures with the ten best%RMSD are shown in Table 1.

### Homology modeling

The 43 sequences highlighted in Figure 4 were considered for prediction of three-dimensional structures using iterative threading assembly refinement algorithm (I-TASSER) (http://zhanglab.ccmb.med.umich.edu/I-TASSER/) [41]. For each sequence, the signal peptides and other domains besides the Cu^2+^-binding catalytic domain were included in the homology modeling. Alignments used for modeling are tabulated in Additional file 2: Table S1. Models obtained with the highest C-score were retained for further analysis. The homology models have been deposited at Model Archive (doi:10.5452/ma-asp8e) [54].

### Structural analysis

Structural comparisons were done using the Combinatorial Extension algorithm [55] implemented in PyMOL (Schrödinger, Portland, OR). Protein surface electrostatics calculations were carried using Adaptive Poisson-Boltzmann Solver (APBS) [56], where an externally generated pdb (P) file with per-atom charge (Q) and radius (R) (PQR file) file was used to calculate the electrostatics. The parameters used were solvent and protein dielectrics of 78.0 and 2.0 respectively, solvent radius of 1.4, and a monovalent ion concentration of 0.15 M. The visualization was depicted in PyMOL with positive and negative molecular surface ranging from -2kT/e to 2kT/e.

### Motif identification

Sequence-based motifs were identified using Multiple Em for Motif Elicitation (MEME) (http://meme.nbcr.net/meme/) [42]. The occurrence of motifs in the sequence was assumed to be distributed either zero or one per sequence. Three motifs were identified for each set of sequences given. The phylogenetic tree of AA10 was divided into four subclades (A to D) based on major phylogenetic clades. For each clade, the motifs were identified using MEME.

### Endnotes

^a^CBM33 has recently been renamed as AA10; likewise GH61 has been renamed as AA9 [11]. These names will be used throughout.

^b^SACTE_3159 from the highly cellulolytic *Streptomyces* sp. SirexAA-E, has also been confirmed to contain Cu^2+^ and have O_2_-dependent cellulose oxidation activity (M. Mbughuni and BG Fox, unpublished data).

## Electronic supplementary material

Additional file 1: Figure S1: Taxonomic diversity of AA10 sequences. AA10 sequences were collected from the CAZy database (608 sequences) and binned into taxonomic categories based on phylum and genus. Three phyla present were Proteobacteria, Firmicutes, and Actinobacteria (central pie chart). Smaller peripheral charts identify the number of sequences within each genus. (ZIP 1 MB)

Additional file 2: Table S1: Sequence alignments used for structural modeling. (XLSX 29 KB)

## Abbreviations

AA: Auxiliary activity
CBM: Carbohydrate-binding module
LPMO: Lytic polysaccharide monooxygenases
GH: Glycoside hydrolase.
